# Transplanting

**Published:** 1878-07

**Authors:** G. R. Thomas

**Affiliations:** Detroit, Mich.


### TRANSPLANTING.

BY G. R. THOMAS, D. D. S., DETROIT, MICH.

With me the operation of replanting teeth has become so common, and the results so uniformly satisfactory, that now I do not hesitate to extract any tooth in the mouth, if the case demands it; and I find the cases that demand it multiplying continually; not that the cases that demand it multiply, but the number of cases I have operated upon, and the observations made, have so strengthened my confidence, that wherein I was once *blind*, I now *see;* not with my mind's eye either; but with the visible eye, I see the end of the root or roots of nearly all my cases of abscessed teeth; and with a record of more than one hundred and fifty cases, with but one single loss, and that in the mouth of a man so timid that he utterly refused to bear the pain which nearly always follows for a few minutes, being compelled to re-extract it in a very short time, convinces me that the operation is not only practical, but decidedly beneficial to both patient and operator; for one sitting is all that I have ever really found necessary to the full and complete restoration of the case. But my object in this communication is not so much to speak of replanting as of transplanting, which I have reason to believe is just as practical so far as the mere matter of reattachment is concerned, as in replanting; and I believe there are but two obstacles in the way of its perfect practicability. First, the difficulty of obtaining the proper teeth at the proper time, and second, the possibility of inoculation; and the latter I regard as by far the most formidable of the two, for I do not regard it as a necessity, that the tooth transplanted should correspond exactly in size and shape to the one extracted; if it is a little too large it may be carefully reduced, or if too small, new osseous deposit will supply the deficiency. But in order to escape the ills that might follow from the second obstacle, I believe we should use the greatest caution. It is not necessary that the transplanted tooth should be a freshly extracted one.

Last winter Mr N. C. a gentleman about thirty-five years of age was under my care for the treatment of his teeth. His front teeth were for the most part, very fine and conspicuous.

The right superior cuspidate had been filled some years previous, filling had been leaking, and having been neglected the crown had wasted away till nothing of the tooth remained when he came under my care save a large hollow root. I made an attempt to pivot, but found the shell too frail.

On the last day of February I extracted the root by means of the screw, and took a corresponding sound tooth that I had removed from a lady's mouth about the same age, just four weeks previous. In preparing for artificial teeth, (it being the only sound tooth in the upper jaw it was thought best to remove it) I opened into the canal and pulp chamber from the apex of the root only, cut the end off one eighth of an inch, it being that much too long; reduced the size somewhat in the centre of the root, it being a trifle larger than the root extracted; filled and placed in position.

The occlusion, shape, and color, were perfect so much so that several dentists who saw the case within a few weeks after the operation, and being told that it was one of the superior six front teeth, and requested to point it out were unable to do so; among whom were Prof. Watling, of Ann

Arbor, and Dr. Haskell, of Chicago, who made the remark "It was well worth coming from Chicago on purpose to see." Perhaps some one is ready to say "it being pulpless it certainly must be discolored." I think such a one would be surprised to see the life like appearance that this tooth retained, and I think the reason is obvious. When we, or nature, through neglect produce death of the pulp in the ordinary way the fluids of the tooth are immediately changed in color, not only from the effect of the poison that may have been used for the purpose of devitalization, but from the leakage that I believe always takes place into the tooth, through the foramen at the apex, after filling the pulp chamber and root canal when the tooth is contained in the socket. I have had occasion to examine the roots of a great many teeth that have been filled, to the apices as has been claimed by those who have a reputation equal to any in the land. But I have never yet found one that was filled thoroughly to the apex. I have occasionally seen one where there was some filling at the apex, and even through the apex, but when opened up and examined it would come far short of a perfect filling. And I believe the imperfect treatment of this apex the point of prolonged trouble in the treatment of abscessed teeth; and this is the advantage gained in the replanting or transplanting operation; the apex is thoroughly filled, leaving no opportunity for the operation of poisonous gases, therefore my opinion is, that if it is found advisable to devitalize, extraction, filling both crown and apex, and replanting is by far the most successful and satisfactory mode of treatment. The perfect filling of the root explains I think the reason why the transplanted tooth has not changed color. In cases where replanting is demanded, except those of immediate destruction of the pulp for the cure of abscess, etc., we invariably find the tooth already discolored.

The two features I wish to make prominent in this case are, that notwithstanding the fact, that the tooth was four weeks in my office, there is no perceptible change in color, and the reattachment is as perfect as though it had been transplanted the same day of extraction.

It has now been three months since the operation, and the patient informs me that he has known no difference, between it and any of his other teeth since the first four weeks.

